# Bladder cancer cells acquire competent mechanisms to escape Fas-mediated apoptosis and immune surveillance in the course of malignant transformation

**DOI:** 10.1054/bjoc.2001.1808

**Published:** 2001-05

**Authors:** F G E Perabo, S Kamp, D Schmidt, H Lindner, G Steiner, R H Mattes, A Wirger, K Pegelow, P Albers, E C Kohn, A von Ruecker, S C Mueller

**Affiliations:** Department of Urology, Rheinische Friedrich-Wilhelms-University Bonn; 53105 Bonn, Germany; Department of Urology, University Hospital Ulm; 89075 Ulm, Germany; Department of Urology, Medical University of Lbeck; 23938 Lbeck, Germany; Institute of Clinical Biochemistry, Rheinische Friedrich-Wilhelms-University Bonn; 53105 Bonn, Germany; Laboratory of Pathology, National Cancer Institute, Bethesda, MD, USA

**Keywords:** bladder cancer, Fas, Fas-ligand, apoptosis, immune escape

## Abstract

Mechanisms of resistance against Fas-mediated cell killing have been reported in different malignancies. However, the biological response of immune escape mechanisms might depend on malignant transformation of cancer cells. In this study we investigated different mechanisms of immune escape in 2 well-differentiated low-grade (RT4 and RT112) and 2 poorly differentiated high-grade (T24 and TCCSUP) bladder cancer cell lines. Fas, the receptor of Fas-ligand, is expressed and shedded by human transitional bladder carcinoma cell lines RT4, RT112, T24 and TCCSUP. Cytotoxicity and apoptosis assays demonstrate that in spite of the Fas expression, poorly differentiated T24 and TCCSUP cells are insensitive towards either recombinant Fas-ligand or agonistic apoptosis-inducing monoclonal antibody against Fas. In poorly differentiated T24 and TCCSUP cell lines we were able to detect marked Fas-ligand protein by flow cytometry and Western blot analysis. In grade 1 RT4 and RT112 cells only minor expression of Fas-ligand possibly because of proteinase action. Fas-ligand mRNA translation or post-translational processing seems to be regulated differentially in the cancer cell lines depending on malignant transformation. In co-culture experiments we show that poorly differentiated cells can induce apoptosis and cell death in Jurkat cells and activated peripheral blood mononuclear cells. This in vitro study suggests that bladder cancer cells can take advantage of different mechanisms of immune evasion and become more competent in avoiding immune surveillance during transformation to higher-grade malignant disease. © 2001 Cancer Research Campaign www.bjcancer.com


*British Journal of Cancer* (2001) **84**(10), 1330–1338
© 2001 Cancer Research Campaign
doi: 10.1054/ bjoc.2001.1808, available online at http://www.idealibrary.com on IDEAL®

http://www.bjcancer.com

### Bladder cancer cells acquire competent mechanisms to escape Fas-mediated apoptosis and immune surveillance in the course of malignant transformation

**FGE Perabo[1], S Kamp[1], D Schmidt[1], H Lindner[1], G Steiner[1], RH Mattes[2], A Wirger[3], K Pegelow[4], P Albers[1], EC Kohn[5], A von Ruecker[4] and SC Mueller[1]**

[1]Department of Urology, Rheinische Friedrich-Wilhelms-University Bonn; 53105 Bonn, Germany; [2]Department of Urology, University Hospital Ulm; 89075 Ulm, Germany; [3]Department of Urology, Medical University of Lübeck; 23938 Lübeck, Germany; [4]Institute of Clinical Biochemistry, Rheinische Friedrich-Wilhelms-University Bonn; 53105 Bonn, Germany; [5]Laboratory of Pathology, National Cancer Institute, Bethesda, MD, USA

**Summary** Mechanisms of resistance against Fas-mediated cell killing have been reported in different malignancies. However, the biological response of immune escape mechanisms might depend on malignant transformation of cancer cells. In this study we investigated different mechanisms of immune escape in 2 well-differentiated low-grade (RT4 and RT112) and 2 poorly differentiated high-grade (T24 and TCCSUP) bladder cancer cell lines. Fas, the receptor of Fas-ligand, is expressed and shedded by human transitional bladder carcinoma cell lines RT4, RT112, T24 and TCCSUP. Cytotoxicity and apoptosis assays demonstrate that in spite of the Fas expression, poorly differentiated T24 and TCCSUP cells are insensitive towards either recombinant Fas-ligand or agonistic apoptosis-inducing monoclonal antibody against Fas. In poorly differentiated T24 and TCCSUP cell lines we were able to detect marked Fas-ligand protein by flow cytometry and Western blot analysis. In grade 1 RT4 and RT112 cells only minor expression of Fas-ligand possibly because of proteinase action. Fas-ligand mRNA translation or post-translational processing seems to be regulated differentially in the cancer cell lines depending on malignant transformation. In co-culture experiments we show that poorly differentiated cells can induce apoptosis and cell death in Jurkat cells and activated peripheral blood mononuclear cells. This in vitro study suggests that bladder cancer cells can take advantage of different mechanisms of immune evasion and become more competent in avoiding immune surveillance during transformation to higher-grade malignant disease. © 2001 Cancer Research Campaign http://www.bjcancer.com

**Keywords**: bladder cancer; Fas; Fas-ligand; apoptosis; immune escape

A prerequisite for cancer cells to expand and survive is the development of strategies for escaping surveillance by the immune system. The Fas/Fas-ligand-system is an important mediator of T cell-and natural killer (NK) cell cytotoxicity. Fas-ligand, a molecule that belongs to the TNF-ligand superfamily (along with TNF, LTα,-β, CD27L, CD30L, CD40L, 4-IBB/CD137, OX40L, TWEAK and TRAIL) is expressed normally on activated NK and T cells (Gruss, 1996; Chicheportiche et al, 1997). It can induce apoptosis in Fas-expressing cells including immune cells. Thereby, Fas-ligand can control various entities of immune regulation including immune response termination (e.g. through peripheral deletion of activated T cells), tolerance acquisition and immune privilege (Kagi et al, 1994; Arase et al, 1995; Nagata, 1997). Fas (APO-1, CD95), the receptor for Fas ligand belongs to the TNF-receptor superfamily and signals cell death after binding with its specific ligand. It is similar in action to other members of this receptor superfamily like TNF-R1, TNF-R2, TRAIL-R1, TRAIL-R2 and TRAMP (Schulze-Osthoff et al, 1998). Cell surface Fas is anchored by a single membrane-spanning domain and is widely expressed on normal and malignant cells (Nagata, 1997). Both Fas and Fas-ligand exist in a membrane bound and a soluble form (Cascino et al, 1995; Liu et al, 1995; Dhein et al, 1995). Soluble Fas (sFas) is generated by alternative mRNA splicing events and to date 4 isoforms of sFas have been described (Cascino et al, 1995; Liu et al, 1995). The most predominant sFas isoform results from the deletion of exon 6 encoding the last 5 amino acid residues of the extracellular domain and 16 of 17 amino acids in the transmembrane domain (Cascino et al, 1995). This isoform has been identified in the supernatants of activated human lymphocytes, several tumour cell lines (Cheng et al, 1994; Natoli et al, 1995; Owen-Schaupp et al, 1995) and in patients with solid tumours (Midis et al, 1996). The soluble form of Fas-ligand is generated by cleavage of a 26 kD TNF-homologous portion of membrane-bound Fas-ligand by a metalloprotease (Tanaka et al, 1995).

The physiological importance of the Fas system was originally thought to be confined to the immune system. Recent evidence demonstrates expression of Fas-ligand not only on NK and T cells (Suda et al, 1993) but also on a variety of non-immunological (Runic et al, 1996; Bamberger et al, 1997; Nagata, 1997) and malignant cells. There it has been demonstrated to be a major factor in the Fas/Fas-ligand based interactions between cytotoxic T lymphocytes and cancer cells in immune escape (Tanaka et al, 1995; Griffith et al, 1996; O'Connell et al, 1996; Runic et al, 1996;

*Received 22 August 2000*
*Revised 22 February 2001*
*Accepted 28 February 2001*

*Correspondence to:* FGE Perabo

Strand et al, 1996; Bamberger et al, 1997; Niehans et al, 1997; Niehans et al, 1997; Shiraki et al, 1997; Ding et al, 1998; Gratas et al, 1998; Ungefroren et al, 1998; Bernstorff et al, 1999). Further strategies of immune escape developed by cancer cells have been proposed. For example, effective utilization of the Fas/Fas-ligand system would also require resistance to Fas-ligand induced signal transduction (Ungefroren et al, 1998). In addition, circulating isoforms of Fas and Fas-ligand do prevent recognition of pathological, i.e. malignant cells by the immune system (Cheng et al, 1994; Hahne et al, 1996; Mitsiades et al, 1998).

In this study we investigated Fas and Fas-ligand expression as well as their sensitivity to Fas-mediated apoptosis in 4 bladder cancer cell lines of different malignant transformation. The possible importance of soluble Fas and Fas-ligand in immune escape was examined by assessing the production of these soluble molecules in the different cancer cell lines. Furthermore the apoptotic potential of (Fas-ligand expressing) bladder cancer cells towards a transformed T cell clone (Jurkat) and stimulated peripheral blood mononuclear cells (PBMC) were analysed by co-culture experiments.

### MATERIAL AND METHODS

### Tumur cell lines and culture conditions

The human transitional cell carcinoma cell lines RT4 (grade 1), RT112 (grade 1), T24 (grade 3) and TCCSUP (grade 4) were obtained from ATCC (Rockville, MD). All tumour cell lines were maintained as adherent cells at 37˚C in 5% $CO_2$ in humidified atmosphere in RPMI 1640 culture medium (Biochrom, Berlin) supplemented with 10% heat-inactivated fetal calf serum (FCS), 1% nonessential amino acids, 2 mM L-glutamine, 100 units $ml^{-1}$ penicillin G, 100 µg $ml^{-1}$ streptomycin and a vitamin solution (Gibco BRL, Life Technologies Inc, Frederick, MD). For experiments, $1 \times 10^6$ tumour cells were seeded in 25 $cm^2$ culture flasks (Costar, Cambridge) and incubated for 48 h to allow maximum confluence thus providing optimal cell–cell contact. Cells were routinely subcultured by trypsination (0.05% Trypsin, 1mM EDTA) (Gibco BRL).

### Antibodies

Inducing anti-Fas mAb (Mouse IgGl, clone DX2) Calbiochem, Cambridge, FITC labeled anti-Fas mAb (Mouse $IgGl_k$, clone DX2) Pharmingen, San Diego, CA; anti-Fas-ligand mAb (Mouse IgGl, clone NOK-1) Pharmingen, San Diego, CA and anti-Fas-ligand mAb (Mouse IgG1, clone H11) Alexis, San Diego, CA were used in the study. Unconjugated IgG1 mAb (Cymbus, Hants, UK) and FITC/PE-conjugated IgG1 mAb (Dako, Denmark) were used as isotype controls.

### Flow cytometric analysis of cell surface Fas and Fas-ligand

The expression of Fas and Fas-ligand on tumour cells was determined by flow cytometry in non-permeabilized cells. Briefly, cells were incubated with matrixmetalloproteinase inhibitor KB8301 (Pharmingen) in normal medium for 24 h and harvested by rapid trypsination to achieve best detachment of cells from the culture flask without damage to membrane integrity (after removal of the culture medium, trypsin was applied for 20 s only, then removed again and cells were incubated for another 5 min without trypsin or medium before adding PBS for washing of cells). KB8301 is added to the cell culture system to increase the levels of mFas-ligand by blocking Fas-ligand cleavage (Kayagaki et al, 1995). $3 \times 10^5$ cells were washed in cold calcium- and magnesium-free PBS and centrifuged at 400 ***g*** for 10 min. Thereafter, the pellet was resuspended in 50 µl FACS buffer (PBS, 5% fetal calf serum, 0.02% sodium citrate) on ice, containing 6 µl of FITC labelled anti-Fas or anti-Fas-ligand-mAb (Clone NOK-1, Pharmingen or clone H-11, Alexis), or 5 µl of FITC conjugated irrelevant mouse IgGl mAb serving as isotype control. After washing in PBS once more, the cells were immediately analysed using a FACS can flow cytometer (Becton Dickinson, San Diego, CA). A minimum of 10 000 events were acquired for each sample.

### Western blot for Fas-ligand

Cells were trypsinized, washed with ice-cold PBS and lysed in lysis buffer (10 mM Tris-Hcl, pH 7.4, 10 mM magnesium chloride, 5 mM EDTA and 1% Triton-X-100) supplemented with 1 mM PMSF (protease inhibitor phenylmethylsulfonyl fluoride) (Boehringer), 200 µg aprotinin (Sigma) and 10 µM leupeptin (Boehringer). Samples containing 100 µg of protein in loading buffer (0.312 M Tris-HC1, pH 6.8, 0.5% bromophenol blue, 10% sodium dodecyl sulfate (SDS), 25% glycerol, 100 mM dithioerythriol) were boiled for 10 min, subjected to SDS-PAGE (12.5%) and transferred to a nitrocellulose transfer membrane (Milipore). Membranes were blocked with 5% non fat dry milk in TBS (50 mM Tris, pH 7.5, 150 mM sodium chloride) and incubated overnight at 4 °C with anti-Fas-ligand-mAb (Alexis, Germany) diluted in 2% TBS Tween 20. Immunological complexes were visualized by enhanced chemiluminiscence (Pierce, Europe BV) using horseradish peroxidase-conjugated goat-antimouse IgG (Dako, Denmark).

### RT-PCR detection of Fas-ligand

#### *RNA isolation and reverse transcriptase (RT)*

Total RNA of $5 \times 10^6$ cells was isolated using a RNA isolation kit based on a modified salt precipitation procedure according to the manufacturer's instructions (Purescript, Gentra Systems Inc, Minneapolis, MS). RNA was precipitated in an equal volume of 2-propanol at-80˚C for 15 min. Precipitates were pelleted at 10 000 ***g*** for 15 min at 4˚C and washed in 75% ethanol. Air-dried pellets were resuspended in RNase-free water. A 50 µl DNase digestion reaction with 25 mM Tris-HCl pH 7.2, 5 mM $MgCl_2$, 0.1 mM EDTA pH 8.0,10 units of DNase, RNase free (Boehringer Mannheim, Germany) and 40 units of RNasin (Promega, Madison, USA) was incubated at 37˚C for 30 min to avoid any DNA contamination of the samples. Total RNA was isolated again and air-dried pellets were resuspended in 50 µl RNase-free water and frozen at –80˚C overnight. The concentration of RNA was estimated by spectrophotometry (OD 260). cDNA synthesis was performed using a MMLV RNase H reverse transcriptase (SuperScript II, Gibco BRL, Life Technologies Inc, Frederick, MA) according to the manufacturer's instructions. Briefly, 1 µg of total RNA template and 1 µl random hexamers (50 µM, Perkin Elmer, Applied Biosystems Division, Foster City, CA) were initially heated to 65˚C for 10 min to reduce secondary structure and quick-chilled on ice. The samples were then incubated for 10 min at 25˚C followed by 60 min at 42˚C with 200 units of

© 2001 Cancer Research Campaign 

Superscript II reverse transcriptase, 500 μM dNTP mix (Pharmacia Biotech, Uppsala, Sweden), 50 mM Tris-HCL pH 8.3, 75 mM KC1, 3 mM $MgCl_2$, 10 mM dithiotreitol and 20 units of RNasin in a 20 μl final volume. The reaction was terminated by heating to 70˚C for 15 min. After 10-fold dilution with DEPC-treated water, samples were stored at −20˚C for subsequent analysis.

### *Fas-ligand cDNA amplification*

Each 20 μl amplification reaction with 10 mM Tris-HCl pH 8.3, 50 mM KCl, 2 mM $MgCl_2$, 200 μM each dNTP, 250 nM each Primer and 0.5 units of AmpliTaq Gold (Perkin Elmer) contained 1 μl of diluted cDNA reaction mixture. Amplifications were carried out using 35 cycles of 94˚C for 30 s, 55˚C for 40s and 72˚C for 50 s after an initial denaturation of 14 min at 95˚C. The primer sequences and amplification product size were as follows: sense, 5′-CAC CTT GGA ATT GTC GTG C-3′ and antisense, 5′-CCT CCA TAT CAG CAG ATC C-3′ (188 bp). To provide a positive control for Fas-ligand expression, PBMC were suspended in RPMI-1640 with 10% autologous serum, stimulated with 5 nM PMA and 1 μM ionomycin for 3 h, and used to prepare cDNA. Control reactions without cDNA were carried out in parallel to ensure that reagents were not contaminated and were consistently negative. Each amplification was carried out 3 times with similar results. PCR products were isolated by gel electrophoresis on 1.75% agarose gels and visualized by staining with GelStar (FMC, Rockland, ME), and their predicted size was verified by comparison to a 100bp ladder (Gibco). Gels were documented with the Gel Doc 1000 video gel documentation system (Bio Rad Laboratories, Hercules, CA).

### Soluble Fas and soluble Fas-ligand ELISA

sFas and sFas-ligand in cell culture supernatants were measured with commercially available enzyme-linked immunosorbent assays (ELISA) ((sFas) Endogene, Woburn, MA/(sFas-ligand) MBL, Naka-ku Nagoya, Japan). In short, a monoclonal antibody recognizing an epitope of Fas and monoclonal antibodies against 2 different epitopes of Fas-ligand (clones 4H9 and 4A5), respectively were precoated onto the walls of microtiter plates. Standards and 1:20 diluted samples were introduced into the wells and immediately the corresponding horseradish peroxidase (HRP) conjugated antibody was added. Following incubation, unbound enzyme-conjugated antibodies were removed by washing and substrate solution was added to the wells. A colored product was formed in proportion to the amount of Fas and Fas-ligand, respectively in the sample. The reaction was stopped with sulfuric acid and the absorbance was measured photometrically by an ELISA reader at 450 nm (SLT, Crailsheim). The concentrations of sFas and sFas-ligand were calculated by means of a corresponding standard curve.

### Cytotoxicity assay

The MTT (Microculture Tetrazolium) assay was used to assess cell viability after exposure to inducing anti-Fas-mAb or recombinant human Fas-ligand (Alexis, San Diego, CA). In brief, trypsinized tumour cells were resuspended in medium at $5 \times 10^5$ cells $ml^{-1}$ after verifying cell viability by trypan blue dye exclusion. 100 μl of cell suspension were distributed into each well of a 96-well flat bottomed microtitre plate and each plate was incubated for 24 h to allow adherent cell growth. Following the incubation, the medium was removed and 200 μl of the different reagent solutions in complete medium and medium control were distributed into each well and incubated for 24, 48 and 72 h. After incubation, 20 μl of the MTT dye working solution (5 mg $ml^{-1}$) (Sigma Chemical Co, St Louis, MO), was added and incubated for 4 h. The supernatant in the wells was decanted and replaced by 200 μl 2-propanol, supplemented with 0.05N HCl to dissolve the reactive dye. The absorbance (A) values of each well were read at 550 nm using an automatic multiwell spectrophotometer (340 ATTC SLT, Crailsheim). The negative control was used for zeroing the absorbance. The growth inhibition percentage was calculated using the background-corrected absorbance as follows: % Growth Inhibition = [(1-A of experimental well)/A of positive control well]*100. Each experimental data point represents average values obtained from 6 replicates, each experiment was performed in duplicate.

### Assessment of apoptosis by FITC-labelled Annexin-V and propidiumiodide

Annexin-V binds to phosphatidylserine (PS), which is exposed to the outer leaflet of the plasma membrane during early apoptosis when the membrane integrity is still maintained. To evaluate the extent and time course of apoptosis after incubating cells with inducing Fas-mAb or rhFas-ligand, Annexin-V and propidiumiodide (PI) were used in double stain technique. Cells were counterstained with PI as a vital dye to distinguish between apoptotic (Annexin V positive, PI negative) and necrotic (Annexin V positive, PI positive) cells, respectively. Following the procedure described above, controls and $3 \times 10^5$ treated cells (all attached cells and detached cells in supernatant) were resuspended in 200 μl of culture medium without phenolred, incubated with 5 μl FITC-conjugated Annexin-V and 10 μl PI for 20 min at room temperature in the dark, and were then immediately analysed by flow cytometry. A minimum of 10 000 events was acquired for each sample.

### Co-culture experiments

Jurkat T leukaemia cells, which express Fas and are sensitive to Fas-ligand induced apoptosis, were a kind gift of Dr J Wessendorf, Department of Dermatology, University of Bonn. Jurkat is a Fas-sensitive cell line of T cell origin that is permanently activated and has been used as a model for activated T cells, with which it shares functional similarities. Furthermore, Jurkat are insensitive to TNF-alpha, another major mediator of apoptotic cell death (Alderson et al, 1995). Peripheral blood mononuclear cells (PBMC) were obtained after Ficoll centrifugation of freshly collected blood of healthy volunteers. PBMC were suspended in RPMI-1640 with 10% autologous serum, stimulated with 5 nM PMA and 1 μM ionomycin for 3 h.

Jurkat cells or PBMC (floating in the medium) were co-cultured with RT4 or T24 bladder cancer cells (growing adherent to the flask bottom) at different ratios (1:10,1:1, 2.5:1 and 10:1) for 24 h. As controls, RT4, T24, Jurkat and PBMC were cultured alone under the same culture conditions. Following co-culture, the floating Jurkat or PBMC were collected from the culture supernatant, then the RT4 or T24 were carefully trypsinized and washed once with PBS. Cells were separately examined for apoptosis by flowcytometry after Annexin-V/PI staining.

© 2001 Cancer Research Campaign

### Statistical analysis

Statistical evaluation of the FACS data was done using the FACScan software, version 'CellQuest' (Becton Dickinson). All experiments were repeated thrice, the results were expressed as the mean ± SD. Statistical analysis of the MTT assay was determined by two-tailed unpaired *t* test. A *P* value of 0.05 or less was considered significant.

### RESULTS

### Expression of cell surface Fas

Flow cytometric analysis of Fas expression was performed on bladder cancer cell lines RT4, RT112, T24, TCCSUP and Jurkat cells as well as on non-activated and activated PBMC. As shown in Figure 1, high Fas expression was found in all the bladder cancer cell lines studied, as well as in Jurkat and both, activated and non-activated PBMC.

### Fas-ligand expression in bladder cancer cell lines

As shown in Figure 2, T24 and TCCSUP cells demonstrated positive staining with the employed antibodies against Fas-ligand, whereas RT4 and RT112 cells only expressed minor (if any) amounts of Fas-ligand. The Fas-ligand antibody from clone H-11 demonstrated a slightly better antigen binding than from clone NOK-1. Jurkat cells and PMA-stimulated PBMC served as a positive control and demonstrated Fas-ligand expression as well. Non-activated PBMC served as negative control (Figure 2).

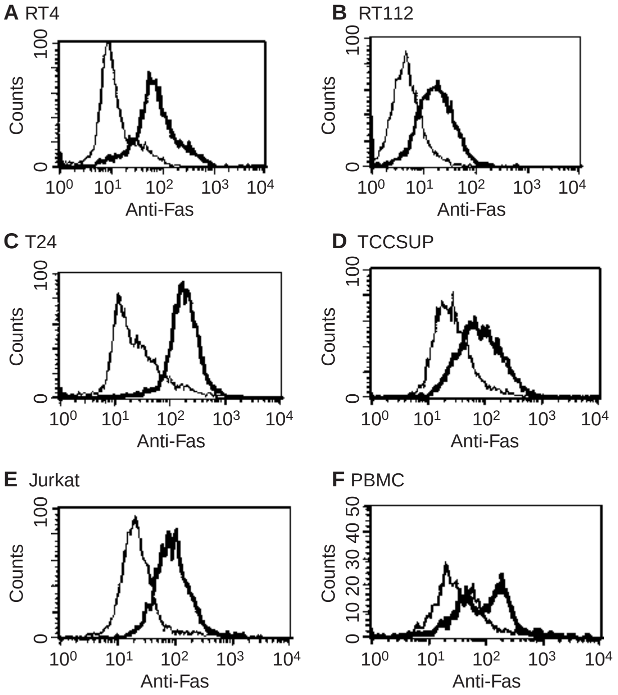

**Figure 1** Cell surface Fas expression on RT4 (**A**), RT112 (**B**), T24 (**C**), SUP (**D**) bladder cancer cells, Jurkat (**E**) and PBMC (**F**); thin line: control with isotype matched irrelevant immunoglobulin, bold line: specific antibody (Fas) expression. $10^6$ cells were stained with FITC-conjugated Fas-mAb (DX-2) and FITC-conjugated IgGl as isotype control and examined by flow cytometric analysis

For verification of Fas-ligand expression we examined whole cell lysates from all cell lines. These lysates were subjected to immunoblotting using the clone H-11 antibody. In T24 and TCCSUP cell lines a band ($M_r$ 26 000) was observed, consistent with the size of a previously reported, protealytically processed form of Fas-ligand (Ungefroren et al, 1998) (Figure 3). Jurkat cells showed a weak positive band for the $M_r$ 26 000 protein, and RT4 and RT112 did not demonstrate any expression of this band, thus suggesting the absence of Fas-ligand in the latter cell lines. We did not succeed in detecting the transmembrane form of the $M_r$ 37 000–40 000 Fas-ligand protein in any cell line. However, the difficulty in demonstrating the presence of this particular protein band in immunoblots has been shown in pancreatic carcinoma (Ungefroren et al, 1998).

To investigate possible differences in the transcription of the Fas-ligand gene, RT-PCR was performed on total RNA extracted from the cell lines. A cDNA fragment of the predicted size (188 bp) could be amplified from all bladder cancer cell lines as well as from Jurkat (Figure 4), the identity of Fas-ligand cDNA was confirmed by sequencing (data not shown).

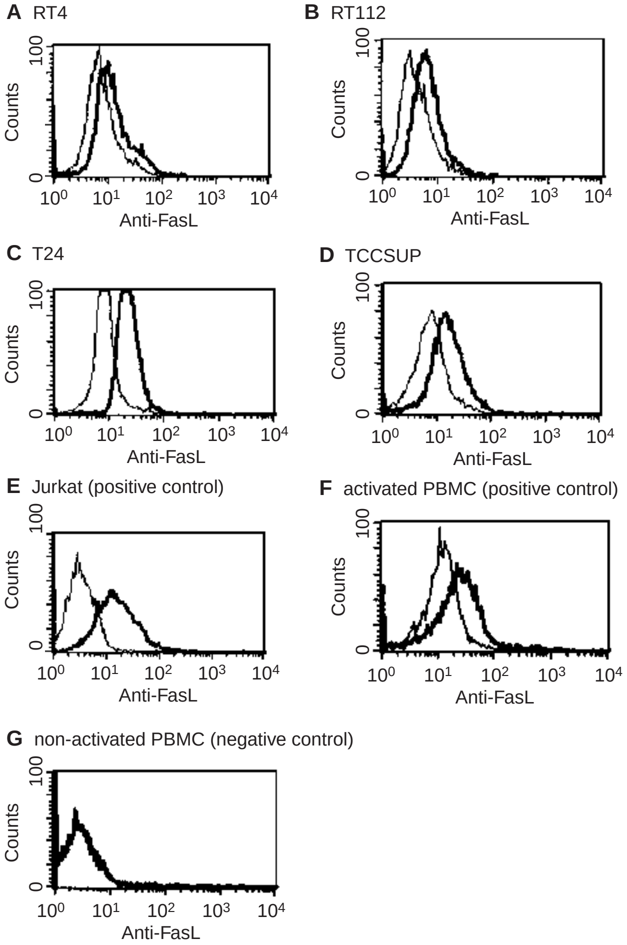

**Figure 2** Flow cytometric analysis of Fas-ligand expression on non-permeabilized human bladder cancer cell lines RT4 (**A**), RT112 (**B**), T24 (**C**), TCCSUP (**D**) bladder cancer cells and Jurkat (**E**) and non-activated PBMC as positive (**F**) and negative (**G**) control cell lines, respectively. Antibody clone H-11 (bold line) or an irrelevant antibody (IgG1, isotype control) for determination of unspecific antibody staining (thin line) was used. RT4 and RT112 express minor Fas-ligand whereas T24, SUP and Jurkat demonstrate specific antigen expression. Non-activated PBMC as negative control demonstrates no Fas-ligand expression (isotype and specific antibody overlaying)

© 2001 Cancer Research Campaign

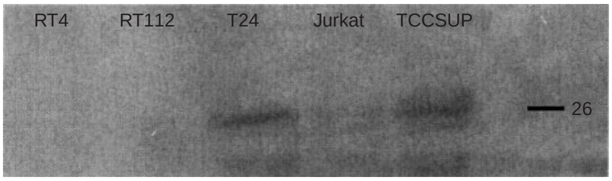

**Figure 3** Immunoblot analysis of Fas-ligand expression in whole cell lysates were subjected to PAGE and blotted onto a nitrocellulose membrane. Fas-ligand in the T24 (lane 3) and TCCSUP (lane 5) cells demonstrate expression of a Mr 28 000 protein. Jurkat control cells demonstrate weak expression (lane 4), and no expression of Fas-ligand in RT4 and RT112 cells (lane 1 and 2)

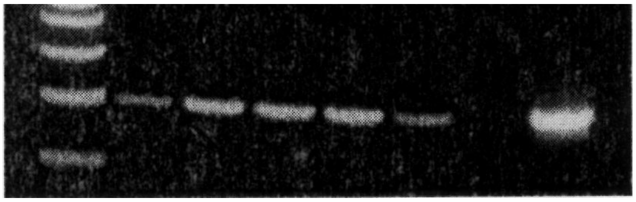

**Figure 4** Bladder cancer cell lines contain Fas-ligand mRNA. Samples of total RNA (1 μg) were reversely transcribed and subjected to 35 PCR cycles as described in 'Materials and Methods'. The amplified products with a size of 188 bp were electrophoresed through a 1.75% agarose gel and stained with GelStar. Line 1: Marker, line 2: RT112, line 3: RT4, line 4: T24, line 5: TCCSUP, line 6: Jurkat, line 7: negative control, line 8: positive control

### Resistance to anti-Fas-mAb and recombinant Fas-ligand

Expression of Fas, though necessary, is not predictive of a biological response to Fas-ligand binding (Owen-Schaub et al, 1994). Some monoclonal antibodies (mAb) against Fas-receptor function as Fas-ligand and can also induce apoptosis (Ogasawara et al, 1993; Nakamura et al, 1997). However, it has recently been suggested that antibody agonists have limited properties in inducing apoptosis (Thilenius et al, 1997), therefore we performed experiments comparing agonistic monoclonal antibody (anti-Fas) and recombinant human Fas-ligand. The effect of these agents on cell viability were investigated with the MTT assay. We found that concentrations of 50 ng $ml^{-1}$, 500 ng $ml^{-1}$ and 5000 ng $ml^{-1}$ of anti-Fas corresponded to the death-inducing potential of 10 ng $ml^{-1}$, 50 ng $ml^{-1}$ and 250 ng $ml^{-1}$ of recombinant Fas-ligand, respectively (data not shown). For practical reasons, we therefore used anti-Fas in further experiments.

Next, we investigated the effects of anti-Fas treatment on cell viability (via MTT assay) and apoptosis induction (via Annexin-V/PI staining) by adding anti-Fas-mAb (1 μg $ml^{-1}$) to the culture

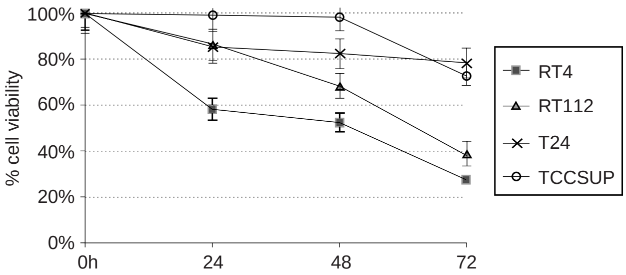

**Figure 5** Comparison of cell viability in RT4, RT112, T24 and TCCSUP cells after 24, 48 and 72 h of incubation time with 1 μg $ml^{-1}$ inducing anti-Fas-mAb as assesed by MTT assay as described in 'Material and Methods'. Difference in viability is matched to the viability of the corresponding untreated cell line. Values expressed as mean ± SD. Statistical difference between RT4/RT112 and T24/TCCSUP after 48 and 72 h ($P < 0.05$)

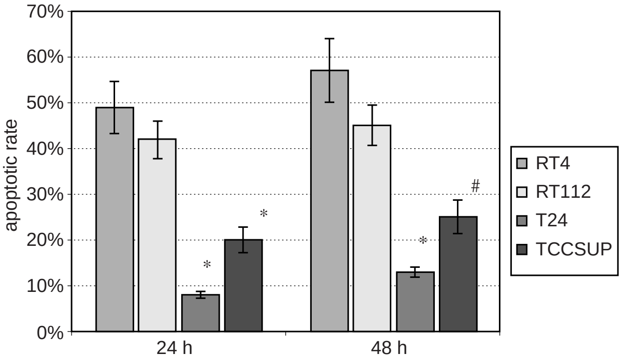

**Figure 6** Comparison of apoptosis induction in bladder cancer cells after 24 and 48 h of incubation time with 1 μg $ml^{-1}$ inducing anti-Fas-mAb as assessed by FACS analysis after Annexin-V/PI staining. All cell lines demonstrated a spontaneous apoptotic rate of cells of <15%. Values expressed as mean ± SD. *$P < 0.01$, #$P < 0.05$

medium. In the MTT assay, RT4 and RT112 cells showed a marked 60–70% reduction in cell viability ($P < 0.05$) after 72 h treatment with anti-Fas. On the other hand, T24 and TCCSUP showed only a limited decrease in cell viability of approximately 30–40% (Figure 5).

Evaluation of apoptosis demonstrated a low spontaneous apoptotic rate of <20% in untreated cell lines. After exposure to anti-Fas-mAb the proportion of apoptotic cells increased up 4-fold in RT4 and RT112 cell lines, whereas in T24 and TCCSUP cell lines, the increase in apoptosis was significantly less ($P < 0.05$) (Figure 6).

### Production of sFas and sFas-ligand by bladder cancer cell lines

Cell culture supernatants with increasing cell counts from $10^3$–$10^6$ cells were analysed by specific ELISAs. Only at a cell count of $>10^6$ cells $ml^{-1}$ we found a measurable sFas concentration in the cell lines (Table 1). We could not detect soluble Fas-ligand in culture supernatants of all cell lines with the employed ELISA kit.

### Interaction of bladder cancer cells with Jurkat and activated PBMC

Having demonstrated that high grade malignant bladder cancer cells like T24 and TCCSUP express Fas-ligand, yet are largely insensitive to Fas-mediated apoptosis, we wanted to see if the Fas-ligand is functional, i.e. if bladder cancer cells can induce apoptosis in Fas-expressing immune cells like Jurkat or activated PBMC. On the other hand, RT112 and RT4, not expressing Fas-ligand, should not be able to induce apoptosis in Jurkat or PBMC. To address this question, co-culture experiments were used.

**Table 1** sFas in cell culture supernatants from $10^3$–$10^7$ cells were repeated determined and analysed by specific ELISA as described in Materials and Methods. Concentrations of sFas were determined by comparison with a standard curve. Values are expressed as median and range

| TCC cell line | sFas (U $ml^{-1}$) $10^6$ cells | sFas (U $ml^{-1}$) $10^7$ cells |
|---|---|---|
| RT112 | 3.1 (1.1–4.5) | 5.1 (1.9–8.3) |
| RT4 | 2.3 (1.3–5.2) | 6.2 (2.4–9.1) |
| T24 | 3.3 (1.9–5.5) | 4.9 (2.1–7.9) |
| TCCSUP | 2.7 (1.3–4.7) | 7.7 (2.6–8.5) |

© 2001 Cancer Research Campaign

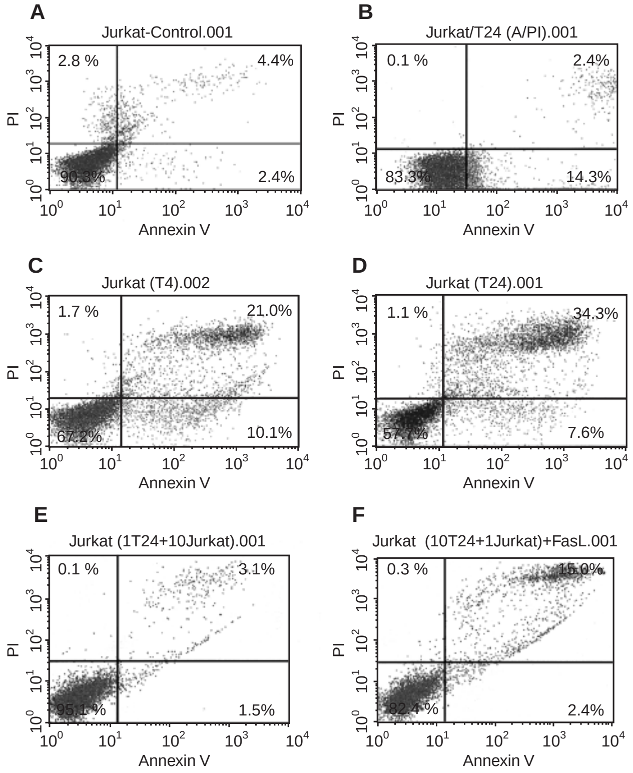

**Figure 7** Flow cytometric analysis with Annexin-V/PI-staining of Fas sensitive Jurkat T leukaemia cells undergoing apoptosis after 24 h coculture with T24. Each Annexin/PI staining was matched to the corresponding autofluorescence of cells. (**A**) Untreated Jurkat cells, (**B**) Jurkat/T24 ratio 1:1, (**C**) Jurkat/T24 ratio 1:25, (**D**) Jurkat/T24 ratio 1:10; (**E**) Jurkat/T24 ratio 10:1, (**F**) Jurkat/T24 ratio 1:10 with 10 μg ml$^{-1}$ neutralizing Fas-ligand-mAb (clone NOK-1). *Dot plot*: Untreated cells were: Annexin-V and PI neg, indicating cells are viable and not undergoing apoptosis; after coculture an increasing percentage of cells were Annexin-V pos and PI pos, indicating cells are undergoing apoptosis

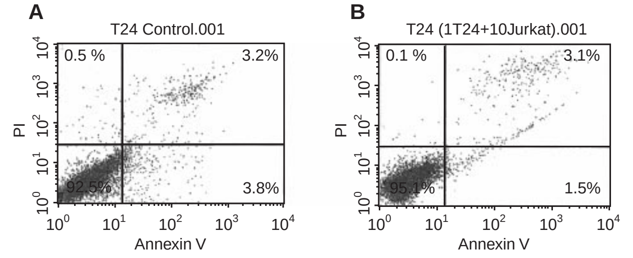

**Figure 8** Flow cytometric analysis with Fas-ligand insensitive T24 cells as target cells co-cultured with 10-fold higher rate of Jurkat. Cells are not undergoing apoptosis after 24 h. Each Annexin/PI staining was matched to the corresponding autofluorescence of cells. (**A**) Untreated T24 cells, (**B**) T24/Jurkat-ratio 1:10

As shown in Figure 7A–D, T24 cells induces apoptosis and cell death in Jurkat cells. With increasing numbers of T24, the rate of apoptotic Jurkat cells increased. At an T24/Jurkat-ratio of 10:1 up to 41.9% apoptosis was seen in Jurkat, whereas in a 10:1 Jurkat/T24-ratio no apoptosis of Jurkat was found (Figure 7E). To confirm, that Jurkat cell killing was mediated by Fas-ligand, experiments with neutralizing anti-Fas-ligand antibody were performed. Thereby, 10 μg ml$^{-1}$ of the clone NOK-1 antibody was added to the co-cultured cells. As shown Figure 7F, the rate of apoptosis was significantly lower in cells treated with a Fas-ligand blocking antibody.

On the other hand, T24 cancer cells were not killed even by high rates (1:10) of Jurkat (Figure 8). The effect of T24 cells on

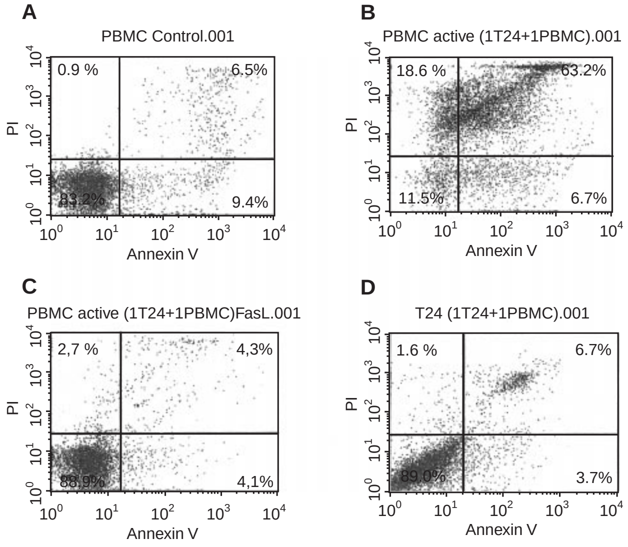

**Figure 9** Fas sensitive activated peripheral blood lymphocytes (PBMC) as target after co-culture with T24 bladder cancer cells (**A**) Untreated activated PBMC, (**B**) T24/PBMC-ratio 1:1, (**C**) T24/PBMC-ratio 1:1 with 10 μg ml$^{-1}$ neutralizing Fas-ligand-mAb (clone NOK-1), (**D**) T24 cells after co-culture with PBMC at a ratio 1:1. Flowcytometric analysis done with Annexin-V/PI-staining activated PBMC was more pronounced. At a ratio of 1:1 a significant increase in apoptosis (69.9%) could be observed compared to the spontaneous apoptotic rate (15.9%), which could be blocked by Fas-ligand antibody (Figure 9A–C). Again, the immune cells (activated PBMC) did not affect T24 viability significantly (Figure 9D).

The lower-grade malignant RT4, lacking Fas-ligand, were not able to induce apoptosis in Jurkat or PBMC, but were also not susceptible to high rates (10:1) of Fas-ligand expressing Jurkat or PBMC cells (data not shown).

### DISCUSSION

Mechanisms of resistance against Fas mediated cell killing have been reported in different malignancies (Dhein et al, 1995; Natoli et al, 1995; O'Connell et al, 1996; Strand et al, 1996; Niehans et al, 1997; Shiraki et al, 1997; Ding et al, 1998; Gratas et al, 1998; Ungefroren et al, 1998; Bernstorff et al, 1999). To date, these studies were performed without taking into consideration that the biological response of immune escape mechanisms might depend on malignant transformation of cancer cells.

We now report that Fas, the receptor of Fas-ligand, is expressed and shedded by human transitional bladder carcinoma cell lines RT4, RT112, T24 and TCCSUP. Treatment of these cell lines with agonistic anti-Fas antibody results in different apoptotic susceptibility in grade 1 (RT4, RT112), grade 3 (T24) and grade 4 (TCCSUP) bladder cancer cell lines. Only the well differentiated cell lines, RT4 and RT112, were sensitive to anti-Fas. We demonstrate, that all cancer cell lines transcribe the Fas-ligand gene, but only poorly differentiated cell lines accumulate substantial amounts of Fas-ligand protein. In co-culture experiments we show that poorly differentiated cells can induce apoptosis and cell death in Jurkat cells and activated peripheral blood mononuclear cells. The cell surface expression of Fas is prerequisite for an effective Fas/Fas-ligand interaction between immune and malignant cells. Lack of Fas receptor expression can be one mechanism of apoptosis resistance (Strand et al, 1996). Since Fas receptor expression is correlated with sensitivity towards Fas-ligand mediated apoptosis (Moers et al, 1999), the down-regulation or lack of Fas receptor expression on cancer cells is one of the mechanisms of immune escape. Finding Fas expression on all bladder cancer cell lines could imply that those cell lines respond to Fas-mediated cell death. However, cytotoxicity and apoptosis assays demonstrate that in spite of Fas expression, poorly differentiated T24 and TCCSUP cells are insensitive towards either recombinant Fas-ligand or agonistic apoptosis-inducing monoclonal antibody against Fas. Thus, the poorly differentiated cells seem to have acquired resistance towards Fas-inducible apoptosis downstream to the Fas-receptor. Decreased susceptibility or resistance to Fas-mediated apoptosis has been reported previously in carcinoma of the liver (Owen-Schaupp et al, 1995), colon (O'Connell et al, 1996) and pancreas (Ungefroren et al, 1998). It has been suggested that cancer cells must be resistant to Fas-signalling not only to escape a cytotoxic attack by Fas-ligand expressing T cells but also to avoid fratricide by Fas-ligand carrying sister cancer cells (Ungefroren et al, 1998).

Several Fas signal-blocking factors or mechanisms may be responsible for this phenomenon. The X-chromosome-linked-inhibitor of apoptosis protein (XIAP) or survivin, a member of the IAP gene family, can block apoptosis induction by Fas through binding specifically to the terminal effector proteases caspase-3 and caspase-7 (Tamm et al, 1998; Yamaguchi et al, 1999). Other factors that may influence Fas signalling include FLICE-inhibitory proteins (FLIPs) (Irmler et al, 1997), bcl-2 influence on the intracellular signalling pathways of Fas (Itoh et al, 1993; Weller et al, 1995) and various components of the ubiquitin-proteasome complex (Wang and Lenardo, 1997), but their individual role in cancer development is not yet clear.

For measuring Fas-ligand expression on bladder cancer cells we employed flow cytometry, immunoblotting and RT-PCR. In poorly differentiated T24 and TCCSUP cell lines we were able to detect marked Fas-ligand protein by flow cytometry and Western blot analysis. In grade 1 RT4 and RT112 cells only minor expression of Fas-ligand protein was found by flow cytometry and Western blot failed to detect the full-length, membrane bound form of Fas-ligand possibly because of metalloproteinase action (Ungefroren et al, 1998; Powell et al, 1999; Gastman et al, 2000). Fas-ligand mRNA expression was found in all bladder cancer cell lines by performing RT-PCR. Therefore, Fas-ligand mRNA translation and/or post-translational processing seems to be regulated differentially in the cancer cell lines. The marked expression of Fas-ligand protein in high-grade T24 and TCCSUP cell lines in comparison to the minor expression in low-grade RT4 and RT112 cells suggests that malignant transformation to higher-grade bladder cancer goes along with (post-)transcriptional events by which Fas-ligand protein production and/or processing is positively supported.

Our finding that T24 and SUP bladder cancer cells express Fas-ligand protein led to co-culture experiments with Jurkat, as a model of activated T cells, and with peripheral blood mononuclear cells (PBMC) to test the functional properties of this molecule as presented by the TCC. Our results show that T24 cells bearing Fas-ligand can effectively kill Jurkat cells and activated PBMC whereas Fas-ligand negative RT4 cells could not. This cell killing is mediated by the Fas-ligand as shown in the experiments where anti-Fas-ligand abrogates the functional property of the Fas-ligand. Together, this suggests that high-grade malignant bladder cancers may be more competent in adopting mechanisms of immune escape than low-grade cancers. On the other hand, Jurkat cells and PBMC, although expressing Fas-ligand themselves, did not induce apoptosis in T24 cells which coincides with the T24 cell resistance to anti-Fas inducible apoptosis. However, Fas sensitive RT4 cells showed no increase in cell death after co-culture with Jurkat and activated PBMC cells. Although induction of apoptosis was expected, repeated experiment did not show this effect. The reason might be biological concentration and regulation differences between apoptosis-inducing anti-Fas-mAb and the Fas-ligand presented on activated immune cells.

The production of soluble Fas and soluble Fas-ligand may add to the immunosuppressive effects of tumours (Hahne et al, 1996) and was tested in all bladder cancer cell lines. Finally, we were able to demonstrate the production of soluble Fas (sFas) but not of soluble Fas-ligand in all bladder cancer cell lines. The importance of this finding for immune escape may be that sFas can antagonize/neutralize cell surface Fas-ligand of T cells and thus decrease their anti-tumour cytotoxic activity (Mizutani et al, 1998). Mizutani et al (1998) reported elevated sFas levels in the serum of patients with bladder cancer and suggested an association of high sFas levels with poor prognosis.

Both sFas-ligand and sFas are released to the extracellular milieu and may, beyond the local site of tumour establishment, induce apoptosis in lymphocytes and Fas-expressing cells as well as diminish immune cell function in potential metastatic sites, thus facilitating tumour cell propagation (Mitsiades et al, 1998). In another study, paraffin-embedded tissues from 37 patients with TCC were analysed by immunohistochemistry to determine Fas and Fas-ligand expression. Fas and Fas-ligand were detected on the cell surface and cytoplasm of 92% of cases, but the expression did not differ with the cytological grade of the TCC. The authors suggest that Fas-ligand may contribute to the immune escape of TCC by killing Fas-bearing lymphocytes and that co-expression of Fas with Fas-ligand also suggests that TCC may have pathways resistant to Fas-mediated autocrine cell suicide (Lee et al, 1999). This observation supports the results of the present study.

In a study by Muschen et al (1999) it was shown that breast cancer cells have an inverse regulation of Fas-ligand and receptor expression during dedifferentiation. In addition, tissue sections showed apoptosis of tumour infiltrating lymphocytes and stromal cell in close proximity to Fas-ligand expressing high-grade breast cancer cells.

Mizutani et al suggested that anti-tumor cytotoxic lymphocytes can coexist with immunoresistant tumour cells. It was reasoned that anti-tumor cytotoxic activity of lymphocytes may be revealed only if the tumour cells are sensitized to killing. The authors investigated the effect of adriamycin (ADR) on the susceptibility of freshly isolated bladder cancer cells to lysis by autologous non-activated peripheral blood lymphocytes (PBL) and tumour-infiltrating lymphocytes (TIL). It was observed that PBL and TIL in patients with bladder cancer exhibited anti-tumor cytotoxic properties, but these properties were not manifest due to development or acquisition of tumour cell resistance. However, the resistance of bladder cancer cells to killing by cytotoxic lymphocytes was overcome if cancer cells were sensitized by subtoxic concentrations of ADR. These findings may suggest that treatment of bladder cancer patients with low doses of ADR may sensitize the cancer cells to killing by autologous circulating and tumour-infiltrating lymphocytes (Mizutani et al, 1999).

In summary, we demonstrated the expression of functional Fas-ligand on high-grade cancer cell lines and their resistance to

© 2001 Cancer Research Campaign

Fas-mediated apoptosis. Furthermore we displayed the production of soluble Fas molecules in all bladder cancer cells. Bladder cancer cells may take advantage of different mechanisms of immune evasion and become more competent in avoiding immune surveillance during transformation to higher-grade malignant disease as found in this in vitro study. The understanding of these multifactorial mechanisms that can determine cancer resistance will hopefully contribute to new strategies in treatment.

### ACKNOWLEDGEMENTS

This work was supported in part by a grant from the A von Krupp and Bohlen-Halbach Stiftung, Essen, Germany.

### REFERENCES

Alderson MR, Tough TW, Davis-Smith T, Braddy S, Falk B, Schooley A, Goodwin RG, Smith CA, Ramsdell F and Lynch DH (1995) Fas ligand mediates activation-induced cell death in human T lymphocytes. *J Exp Med* **181**: 71–77

Arase H, Arase N and Saito T (1995) Fas-mediated cytotoxicity by freshly isolated natural killer cells. *J Exp Med* **181**: 1235–1238

Bamberger AM, Schulte HM, Thuneke I, Erdmann I, Bamberger CM and Asa SL (1997) Expression of the apoptosis-inducing Fas ligand (FasL) in human first and third trimester placenta and choriocarcinoma cells. *J Clin Endocrinol Metab* **82**: 3173–3175

Bernstorff W, Spanjaard RA, Chan AK, Lockhart DC, Sadanaga N, Wood I, Peiper M, Goedegebuure PS and Eberlein TJ (1999) Pancreatic cancer cells can evade immune surveillance via nonfunctional Fas (APO-1/CD95) receptors and aberrant expression of functional Fas ligand. *Surgery* **125**: 73–84

Cascino I, Fiucci G, Papoff G and Ruberti G (1995) Three functional soluble forms of the human apoptosis-inducing Fas-molecule are produced by alternative splicing. *J Immunol* **154**: 2706–2713

Cheng J, Zhou T, Liu C, Shapiro JP, Brauer MJ, Kiefer MC, Barr PJ and Mountz JD (1994) Protection from Fas-mediated apoptosis by a soluble form of the Fas molecule. *Science* **263**: 1759–1762

Chicheportiche Y, Bourdon PR, Xu H, Hsu YM, Scott H, Hession C, Garcia I and Browning JL (1997) TWEAK, a new secreted ligand in the tumor necrosis factor family that weakly induces apoptosis. *J Biol Chem* **272**: 32401–32410

Crowe DL, Boardman ML and Fong KS (1998) Anti-Fas antibody differentially regulates apoptosis in Fas ligand resistant carcinoma lines via the caspase 3 family of cell death proteases but independently of bcl2 expression. *Anticancer Res* **18**: 3163–3170

Dhein J, Walczak H, Bäumle D, Debatin KM and Krammer PH (1995) Autocrine T-cell suicide mediated by APO-1/(FasCD95). *Nature* **373**: 438–440

Ding EX, Hizuta A, Morimoto Y, Tanida T, Hongo T, Ishii T, Yamano T, Fujiwara T, Iwagaki H and Tanaka N (1998) Human colon cancer cells express the functional Fas ligand. *Res Commun Mol Pathol Pharmacol* **101**: 13–24

Gastman BR, Yin XM, Johnson DE, Wieckowski E, Wang GO, Watkins SC and Rabinovich H (2000) Fas ligand is expressed on human squamous cell carcinomas of the head and neck, and it promotes apoptosis of T lymphocytes. *Cancer Res* **59**: 5356–5364

Gratas C, Tohma Y, Van Meir EG, Klein M, Tenan M, Ishii N, Tachibana O, Kleihues P and Ohgaki H (1997) Fas ligand expression in human glioblastoma cell lines and primary astrocytic brain tumors. *Brain Pathol* **7**: 863–869

Gratas C, Tohma Y, Barnas C, Taniere P, Hainaut P and Ohgaki H (1998) Up-regulation of Fas (APO-1/CD95) ligand and down-regulation of Fas expression in human esophageal cancer. *Cancer Res* **58**: 2057–2062

Griffith TS, Yu X, Herndon JM, Green DR and Ferguson TA (1996) CD95-induced apoptosis of lymphocytes in an immune privileged site induces immunological tolerance. *Immunity* **5**: 7–16

Gruss HJ (1996) Molecular, structural, and biological characteristics of the tumor necrosis factor ligand superfamily. *Int J Clin Lab Res* **26**: 143–159

Hahne M, Rimoldi D, Schröter M, Romero P, Schreier M, French LE, Schneider P, Bornard T, Fontana A, Lienard D, Cerottini JC and Tschopp J (1996) Melanoma cell expression of Fas (APO-1/CD95) ligand: Implication for tumor immune escape. *Science* **274**: 1363–1366

Irmler M, Thome M, Hahne M, Schneider P, Hofmann K, Steiner V, Bodmer JL, Schroter M, Burns K, Mattmann C, Rimoldi D, French LE and Tschopp J (1997) Inhibition of death receptor signals by cellular FLIP. *Nature* **388**: 190–195

Itoh N, Tsujimoto Y and Nagata S (1993) Effect of bcl-2 on Fas-antigen mediated cell death. *J Immunol* **151**: 621–6227

Kagi D, Vignaux F, Ledermann B, Burki K, Depraetere V, Nagata S, Hengartner H and Golstein P (1994) Fas and perforin pathways as major mechanisms of T cell-mediated cytotoxicity. *Science* **265**: 528–530

Kayagaki NA, Kawasaki T, Ebata H, Ohmoto S, Ikeda S, Yoshino K, Okumura K and Yagita H (1995) Metalloproteinase-mediated release of human Fas ligand. *J Exp Med* **182**: 1777–1783

Klas C, Debatin KM, Jonker RR and Krammer PH (1993) Activation interferes with the APO-1 pathway in mature human T cells. *Int Immunol* **5**: 625–630

Lee SH, Lee JY, Park WS, Kim SY, Jang JJ and Yoo NJ (1999) Transitional cell carcinoma expresses high levels of Fas ligand in vivo. *BJU Int* **83**: 698–702

Liu C, Cheng J and Mountz J (1995) Differential expression of human Fas mRNA species upon peripheral blood mononuclear cell activation. *J Biochem* **310**: 957–963

Midis GP, Shen Y and Owen-Schaupp LB (1996) Elevated soluble Fas (sFas) in nonhematopoetic human malignancy. *Cancer Res* **56**: 3870–3874

Mitsiades N, Poulaki V, Kotoula V, Leone A and Tsokos M (1998) Fas ligand is present in tumors of the Ewing's sarcoma family and is cleaved into a soluble form by a metalloproteinase. *Am J Pathol* **153**: 1947–1956

Mizutani Y, Yoshida O and Bonavida B (1998) Prognostic significance of soluble Fas in the serum of patients with bladder cancer. *J Urol* **160**: 571–576

Mizutani Y, Yoshida O and Miki T (1999) Adriamycin-mediated potentiation of cytotoxicity against freshly isolated bladder cancer cells by autologous non-activated peripheral blood lymphocytes and tumor infiltrating lymphocytes. *J Urol* **62**: 2170–2175

Moers C, Warskulat U, Muschen M, Even J, Niederacher D, Josien R, Koldovsky U, Beckmann MW and Haussinger D (1999) Regulation of CD95 (Fas/APO-1) ligand and receptor expression in squamous cell carcinoma by interferon-γ and cisplatin. *Int J Cancer* **80**: 564–572

Muschen M, Moers C, Warskulat U, Niederacher D, Betz B, Even J, Lim A, Josien R, Beckmann MW and Haussinger D (1999) CD95 ligand expression in dedifferentiated breast cancer. *J Pathol* **189**: 378–386

Nagata S (1997) Apoptosis by death factor. *Cell* **88**: 355–365

Nakamura S, Takeshima M, Nakamura Y, Ohtake S and Matsuda T (1997) Induction of apoptosis in HL60 leukemic cells by anticancer drugs in combination with anti-Fas monoclonal antibody. *Anticancer Res* **17**: 173–179

Natoli G, Ianni A, Costanzo A, De Petrillo G, Ilari I, Chirillo P, Balsano C and Levero M (1995) Resistance to Fas-mediated apoptosis in human hepatoma cells. *Oncogene* **10**: 1157–1164

Niehans GA, Brunner T, Frizelle SP, Liston JC, Salerno CT, Knapp DJ, Green DR and Kratzke RA (1997) Human lung carcinoma express Fas ligand. *Cancer Res* **57**: 1007–1012

O'Connell J, O'Sullivan GC, Collins JK and Shanahan F (1996) The Fas counterattack: Fas-mediated T cell killing by colon cancer cells expressing Fas ligand. *J Exp Med* **184**: 1075–1082

Ogasawara J, Watanabe-Fukunaga R, Adachi M, Matsuzaw A, Kasugai T, Kitamura Y, Itoh N, Suda T and Nagata S (1993) Lethal effect of the anti-Fas-antibody in mice. *Nature* **364**: 806–809

Owen-Schaub LB, Radinsky R, Kruzel E, Berry K and Yonehara S (1994) Anti-Fas on non-hematopoetic tumors: levels of Fas/APO-1 and bcl-2 are not predicive of biological responsiveness. *Cancer Res* **54**: 1580–1586

Owen-Schaub LB, Angelo LS, Radinsky R, Ware CF, Gesener TG, and Bartos DP (1995) Soluble Fas/APO-1 in tumor cells: a potential regulator of apoptosis? Cancer Lett **94**: 1–8

Powell WC, Fingleton B, Wilson CL, Boothby M and Matrisian LM (1999) The metalloproteinase matrilysin proteolytically generates active soluble Fas ligand and potentiates epithelial cell apoptosis. *Curr Biol* **9**: 1441–1447

Runic R, Lockwood CJ, Ma Y, Dipasquale B and Guller S (1996) Expression of Fas ligand by human cytotrophoblasts: implications in placentation and fetal survival. *J Clin Endocrinol Metab* **81**: 3119–3122

Schulze-Osthoff K, Ferrari D, Los M, Wesselborg S and Peter ME (1998) Apoptosis signaling by death receptors. *Eur J Biochem* **254**: 439–459

Shiraki K, Tsuji N, Shioda T, Isselbacher KJ and Takahashi H (1997) Expression of Fas ligand in liver metastases of human colonic adenocarcinomas. *Proc Natl Acad Sci USA* **94**: 620–625

Strand S, Hofmann WJ, Hug H, Müller M, Otto G, Strand D, Mariani SM, Stremmel W, Krammer PH and Galle PR (1996) Lymphocyte apoptosis induced by CD95 (APO-1/Fas) ligand expressing tumor cells – A mechanism of immune evasion? *Nature Med* **12**: 1361–1366

Suda T, Takahashi T, Golstein P and Nagata S (1993) Molecular cloning and expression of the Fas-ligand, a novel member of the tumor necrosis factor family. *Cell* **75**: 1169–1178

*© 2001 Cancer Research Campaign*

Suda T, Hashimoto H, Tanaka M, Ochi T and Nagata S (1997) Membrane Fas ligand kills human peripheral blood T lymphocytes and soluble Fas ligand blocks the killing. *J Exp Med* **186**: 2045–2050

Tamm I, Wang Y, Sausville E, Scudiero DA, Vigna N, Oltersdorf T and Reed JC (1998) IAP-family protein survivin inhibits caspase activity and apoptosis induced by Fas (CD95), Bax, caspases, and anticancer drugs. *Cancer Res* **58**: 5315–5320

Tanaka M, Suda T, Takahashi T and Nagata S (1995) Expression of the functional soluble form of human Fas ligand in activated lymphocytes. *EMBO* **14**: 1129–1135

Thilenius AR, Braun K and Russel JH (1997) Agonist antibody and Fas ligand mediate different sensitivity to death in the signaling pathways of Fas and cytoplasmic mutants. *Eur J Immunol* **27**: 1108–1114

Ungefroren H, Voss M, Jansen M, Roeder C, Henne-Bruns D, Kremer B and Kalthoff H (1998) Human pancreatic adenocarcinomas express Fas and Fas Ligand yet are resistant to Fas-mediated apoptosis. *Cancer Res* **58**: 1741–1749

Von Reyher U, Sträter J, Kittstein W, Gschwendt M, Krammer PH and Möller P (1998) Colon carcinoma cells use different mechanisms to escape CD95-mediated apoptosis. *Cancer Res* **58**: 526–534

Wang J and Lenardo MJ (1997) Molecules involved in cell death and peripheral tolerance. *Curr Opin Immunol* **9**: 818–825

Weller M, Malipiero U, Aguzzi A, Reed JC and Fontana A (1995) Protooncogene bcl-2 gene transfer abrogates Fas/APO-1 antibody-mediated apoptosis of human malignant glioma cells and conferes resistance to chemotherapeutic drugs and therapeutic irradiation. *J Clin Invest* **95**: 2633–2643

Yamaguchi K, Nagai S, Ninomiya-Tsuji J, Nishita M, Tamai K, Irie K, Ueno N, Nishida E, Shibuya H and Matsumoto K (1999) XIAP, a cellular member of the inhibitor of apoptosis protein family, links the receptors to TAB1-TAK1 in the BMP signaling pathway. *EMBO* **18**: 179–187

*© 2001 Cancer Research Campaign*
